# Do Organohalogen Contaminants Contribute to Histopathology in Liver from East Greenland Polar Bears (*Ursus maritimus*)?

**DOI:** 10.1289/ehp.8038

**Published:** 2005-07-05

**Authors:** Christian Sonne, Rune Dietz, Pall S. Leifsson, Erik W. Born, Robert J. Letcher, Maja Kirkegaard, Derek C. G. Muir, Frank F. Riget, Lars Hyldstrup

**Affiliations:** 1 Department of Arctic Environment, National Environmental Research Institute, Roskilde, Denmark; 2 Department of Veterinary Basic Sciences, and; 3 Department of Veterinary Pathobiology, Royal Veterinary and Agricultural University, Frederiksberg, Denmark; 4 Greenland Institute of Natural Resources, Nuuk, Greenland; 5 National Wildlife Research Centre, Canadian Wildlife Service, Environment Canada, Carleton University, Ottawa, Ontario, Canada; 6 National Water Research Institute, Environment Canada, Burlington, Ontario, Canada; 7 University Hospital of Hvidovre, Hvidovre, Denmark

**Keywords:** bile duct proliferation, chlordanes, dichlorodiphenyltrichloroethane, dieldrin, East Greenland, HCB, hexacyclohexanes, Ito cells, lipid granulomas, liver, mononuclear cell infiltrations, polar bear, polybrominated diphenyl ethers, polychlorinated biphenyls, portal fibrosis, ∑DDT, ∑HCH, ∑PBDE, ∑PCB, *Ursus maritimus*

## Abstract

In East Greenland polar bears (*Ursus maritimus*), anthropogenic organohalogen compounds (OHCs) (e.g., polychlorinated biphenyls, dichlorodiphenyltrichloroethane, and polybrominated diphenyl ethers) contributed to renal lesions and are believed to reduce bone mineral density. Because OHCs are also hepatotoxic, we investigated liver histology of 32 subadult, 24 adult female, and 23 adult male East Greenland polar bears sampled during 1999–2002. Light microscopic changes consisted of nuclear displacement from the normal central cytoplasmic location in parenchymal cells, mononuclear cell infiltrations (mainly portally and as lipid granulomas), mild bile duct proliferation accompanied by fibrosis, and fat accumulation in hepatocytes and pluripotent Ito cells. Lipid accumulation in Ito cells and bile duct hyperplasia accompanied by portal fibrosis were correlated to age, whereas no changes were associated with either sex or season (summer vs. winter). For adult females, hepatocytic intracellular fat increased significantly with concentrations of the sum of hexachlorocyclohexanes, as was the case for lipid granulomas and hexachlorobenzene in adult males. Based on these relationships and the nature of the chronic inflammation, we suggest that these findings were caused by aging and long-term exposure to OHCs. Therefore, these changes may be used as biomarkers for OHC exposure in wildlife and humans. To our knowledge, this is the first time liver histology has been evaluated in relation to OHC concentrations in a mammalian wildlife species, and the information is important to future polar bear conservation strategies and health assessments of humans relying on OHC-contaminated food resources.

In rats and mink, several acute studies of polychlorinated biphenyls (PCBs) have associated these compounds with hepatotoxicity (Bergman et al. 1992; Bruckner et al. 1974; Chu et al. 1994; Jonsson et al. 1981; Kelly 1993; Kimbrough et al. 1971; MacLachlan and Cullen 1995; Parkinson 1996). Specifically in the liver, acute organohalogen compound (OHC) toxicity is mediated through sub-cellular toxicity, leading to impaired ATP, protein synthesis, and other changes (Kelly 1993; Parkinson 1996), and chronic exposure also may affect endocrine homeostasis via up-regulation of cytochrome P450 isozymes (e.g., CYP1A and CYP1B) (Boon et al. 1992; Lin et al. 2003; van Duursen et al. 2003; Wong et al. 1992).

In marine wildlife, chronic exposure to organohalogen compounds [OHCs; e.g., PCBs, dichlorodiphenyltrichloroethane (DDT), and polybrominated diphenyl ethers (PBDEs)] has been associated with toxic effects on several organ systems (Bergman 1999; Bergman and Olsson 1985; Bergman et al. 2001; Schumacher et al. 1993). However, histologic liver changes associated with high environmental levels of OHCs in wildlife have been investigated only in birds, such as cormorants (*Phalacrocorax carbo*) (Fabczak et al. 2000), and fish, such as common bream (*Abramis brama*) (Koponen et al. 2001), but never in marine or terrestrial mammals.

Polar bears are the most OHC-contaminated species in the Arctic, and those from East Greenland and Svalbard (Norway) carry the most contaminants because of their reliance on OHC-polluted blubber, mainly from ringed seal (*Phoca hispida*) and bearded seal (*Erignathus barbatus*), contaminanted by OHCs originating from lower-latitude airborne pollution [Arctic Monitoring and Assessment Programme (AMAP) 2004; de March et al. 1998; Ramsay and Stirling 1988]. At Svalbard, recent studies of PCBs and organochlorine (OC) pesticides in polar bears have indicated negative associations with plasma testosterone (males), progesterone (females), cortisol (both sexes), retinol (both sexes), and thyroxine hormone (both sexes) (Braathen et al. 2004; Haave et al. 2003; Oskam et al. 2003, 2004; Skaare et al. 2001). Additionally, high levels of PCBs/OC pesticides were associated with low levels of IgG in the Svalbard bears, suggesting possible immunotoxic effects (Bernhoft et al. 2000; Lie et al. 2004, 2005). In East Greenland polar bears, OHCs are believed to reduce bone mineral density (BMD) and to be a cofactor in the development of renal lesions and splenic changes (Kirkegaard et al. 2005; Sonne et al. 2004, in press). To determine if OHCs are also a cofactor in hepatotoxicity, liver tissue histology was examined in 79 East Greenland polar bears sampled during the subsistence hunt from 1999 to 2002, and liver histology was compared with individual OHC adipose tissue levels in 65 of the bears. These new results are intended to fill part of the existing knowledge gap in understanding the significance, nature, and effects of chronic environmental OHC exposure.

## Materials and Methods

### Sampling.

All polar bear samples were collected from January through September by local subsistence hunters in the Scoresby Sound area in central East Greenland (69°00′N to 74°00′N) during 1999–2002. A tissue subsample from the periphery of a randomly chosen liver lobe was collected from 79 individuals and fixed in a phosphate-buffered formaldehyde/alcohol solution (3.5% formaldehyde, 86% ethanol, and 10.5% H_2_O), which prevented freeze damage. In addition, sternal subcutaneous adipose tissue was sampled from 65 of the individuals for OHC analyses and stored in separate poly-ethylene plastic bags until arrival at the laboratory in Roskilde, where they were transferred into rinsed [acetone (Supra solv. 1.00012), *n*-hexane (Unisolv 1.04369) both from Merck, KGaA, Darmstadt, Germany] glass containers, and covered with aluminum foil in between the sample and the plastic lid. All samples were taken < 12 hr postmortem and preserved frozen during the hunt and later kept at −20°C before preparation and examination at the veterinary pathology laboratory in Copenhagen, Denmark (histology); GLIER, Windsor, Ontario, Canada (organochlorines); and NWRI, Burlington, Ontario, Canada (PBDEs).

### Age estimation.

The age determination was carried out by counting the cementum growth layer groups of the lower left incisor (I_3_) after decalcification, thin sectioning (14 μm), and staining (toluidine blue) using the method described by Dietz et al. (1991) and Hensel and Sorensen (1980). When necessary, the individuals were categorized as adult males (≥ 6 years of age), adult females (≥ 5 years of age), and subadults (those remaining) (Rosing-Asvid et al. 2002). In the evaluation of sex difference in the prevalence of histologic liver changes, bears were categorized as old at ≥ 15 years of age based on Derocher and Stirling (1994).

### Histology.

The liver tissue was trimmed, processed conventionally, embedded in paraffin, sectioned at about 4 μm, and stained with hematoxylin (aluminum-hematein) and eosin (H&E) and periodic acid-Schiff for routine diagnostics; Van Gieson and Masson Trichrome to detect fibrous tissue (collagen); Best’s carmine to demonstrate glycogen storage; Sudan III to detect lipid (frozen tissue); and Perls’ Prussian blue reaction and Schmorl technique for detecting hemosiderin and lipofuscin pigments, respectively (Bancroft and Stevens 1996; Lyon et al. 1991).

We evaluated six histologic changes and grouped them semiquantitatively as follows:

- Portal mononuclear cell infiltrations: absent, unifocally, multifocally, or diffuse
- Random mononuclear cell infiltrations: absent, unifocally, multifocally, or diffuse
- Lipid granulomas: average number in five fields at 10 × magnification
- Hepatocytic intracellular fat: absent, foamy, multifocal macrovesiculary, or diffuse macrovesiculary
- Visible Ito cells: average number in five fields at 20 × magnification
- Mild multifocal bile duct hyperplasia accompanied by portal fibrosis: absent or present.

For each histologic change, the degree of change was measured as follows:

- Portal mononuclear cell infiltrations: mild (unifocally), moderate (multifocally), severe (diffuse)
- Random cell infiltrations: mild (< 1), moderate (1–3), severe (> 3)
- Lipid granulomas: mild (< 1), moderate (1 to < 2), severe (2–5)
- Hepatocytic intracellular fat: mild (foamy), moderate (multifocal macrovesiculary), severe (diffuse macrovesiculary)
- Ito cells: mild (< 10), moderate (10 to < 50), severe (50–200).

### Analyses of OHCs.

Polar bear subcutaneous adipose tissue samples (*n* = 65) were analyzed for PCBs, DDTs, chlordanes (CHLs), dieldrin, hexacyclohexanes (HCHs), and hexachlorobenzene (HCB) according to Dietz et al. (2004) and Sandala et al. (2004) at the Great Lakes Institute for Environmental Research (University of Windsor, Windsor, Ontario, Canada). An external standard quantification approach used for PCBs and OC pesticides in the subcutaneous adipose tissues was based on peak area of the gas chromatography-electron capture detection response, which is described in detail by Luross et al. (2002).

Briefly, ∑PCB is the sum of the concentrations of the 51 individual or coeluting PCB congeners (if detected), given by International Union of Pure and Applied Chemistry (IUPAC) number: 31/28, 52, 49, 44, 42, 64/71, 74, 70, 66/95, 60, 101/84, 99, 97, 87, 110, 151, 149, 118, 146, 153, 105, 141, 179, 138, 158, 129/178, 182/187, 183, 128, 174, 177, 171/202/156, 200, 172, 180, 170/190, 201, 203/196, 195, 194, and 206. ∑DDT is the sum of 4,4′-DDT, 4,4′-dichlorodiphenyl-dichloroethane (DDD), and 4,4′-dichlorodiphenyldichloroethylene (DDE). ∑HCH is the sum of the α-, β-, and γ-hexachlorocyclohexane. ∑CHL is the sum of oxychlordane, *trans*-chlordane, *cis*-chlordane, *trans*-nonachlor, *cis*-nonachlor, and heptachlor epoxide. OHC fractions were subsequently sent to the National Water Research Institute for determination of brominated diphenyl ether (PBDE) flame retardants. PBDEs (*n* = 65) were determined by electron capture negative ion (low resolution) mass spectroscopy using an external standard. Briefly, ∑PBDE is the sum of the concentrations of the 35 individual or coeluting congeners (if detected), given by IUPAC number: 10, 7, 11, 8, 12/13, 15, 30, 32, 28/33, 35, 37, 75, 71, 66, 47, 49, 77, 100, 119, 99, 116, 85, 155/126, 105, 154, 153, 140, 138, 166, 183, 181, and 190. Gas chromatographic conditions for the PBDEs were as described by Luross et al. (2002).

### Statistics.

The statistical analyses were performed with SAS statistical software (version 8, and Enterprise Guide, version 1; SAS Institute, Cary, NC, USA); the level of significance was set at *p* ≤ 0.05, and levels of significance at 0.05 < *p* ≤ 0.10 were considered a trend. The OHC data were log-transformed (base *e*) before the analyses in order to meet the assumption of normality and homogeneity of the variance.

For each specific histologic liver change, we performed a one-way analysis of variance (ANOVA) to test for differences in mean age between individuals with and without that specific histologic liver change (Table 1). In the case of hepatocytic lipid, we compared foamy cytoplasm with macrovesicular lipid. Furthermore, we tested whether there was a relationship between sex or season (summer, 1 June through 30 September; winter, 1 October through 31 May), and histologic liver changes using a chi-square test. In the case of Ito cells and bile duct hyperplasia accompanied by portal fibrosis, we performed the chi-square test within subadult, adult, and old bears to determine age dependency. The chi-square test was also used to test the relationship between Ito cells and fatty granulomas.

We then performed a one-way ANOVA to test for differences in mean concentrations of each group of OHCs (PCBs, DDTs, CHLs, dieldrin, HCHs, HCB, and PBDEs) between subadults, adult females, and adult males (Table 2). The results were then evaluated from Tukey’s post hoc test. In order to test the relationship between concentrations of OHCs and age, we used a linear regression model for subadults, adult females, and adult males.

Finally, we tested the relationship between the concentrations of each group of OHCs (PCBs, DDTs, CHLs, dieldrin, HCHs, HCB, and PBDEs, respectively) and each histologic liver change (absent vs. present) by an analysis of covariance (Table 3). This was conducted for each of the three age/sex groups using OHC concentration as the dependent variable, age as the covariable, and histologic liver change as the class variable, including their first-order interaction links (age × histologic liver change). The statistical analyses were performed separately on subadults, adult females, and adult males in cases of CHLs, dieldrin, HCHs, and HCB, because the age relationships and/or concentrations differed among these three age/sex groups. In the case of lipid granulomas, the relationship to OHCs was analyzed based on the presence or absence of Ito cells. After a successive reduction of non-significant interactions, judged from the type III sum of squares (*p* ≤ 0.05), the significance of each of the remaining factors was evaluated from the final model least-square mean.

## Results

**Figure 1:**
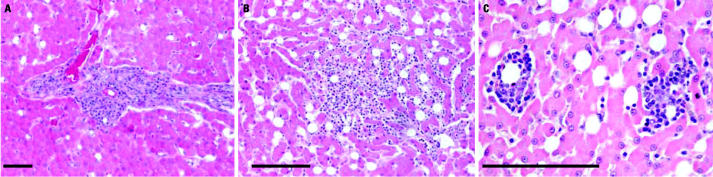
Liver tissue stained with H&E showing portal mononuclear cell infiltration in a 3.5-year-old (subadult) female (*A*; 10×), random mononuclear cell infiltration in a 20-year-old female (*B*; 20×), and lipid granulomas in a 16-year-old female (*C*; 40×) in liver tissue stained with H&E. Note the abnormal localization of the hepatocytic nuclei in (*C*). Bars = 50 μm.

We studied a total of 79 free-ranging East Greenland polar bears (24 subadults, 24 adult females, 22 adult males, 4 old females, and 5 old males), collected from 1999 through 2002 (Table 1). No background data describing the general liver histology of free-ranging polar bears were available in the scientific literature. The morphology of the liver tissue was similar to other carnivorous species; however, interlobular fibrous septa were lacking as in other ursid species (Frappier 1998; Heier et al. 2003, in press; Kelly 1993; Leighton et al. 1988; MacLachlan and Cullen 1995; Prunescu et al. 2003). Kupffer cells, located in the space of Disse, tested positive for hemosiderin (iron pigments) (Lyon et al. 1991), and hepatocytes tested positive for deposits compatible with glycogen (Bancroft and Stevens 1996). In all individuals, parenchymal cells exhibited nuclear displacement toward the cell membrane (Figure 1) (Sato et al. 2001).

### Mononuclear cell infiltrations and lipid granulomas.

We found portal mononuclear cell infiltrations (lymphocytes, macrophages, and neutrophils), as described by Kelly (1993) and MacLachlan and Cullen (1995), in 18% of the animals and multifocally mononuclear cell infiltrations in 12% of the bears examined (Table 1, Figure 1). Additionally, we detected lipid granulomas, also described by these authors, in 76% of the animals. None of these three cell infiltration types was related to age, sex, or season (all, *p* > 0.05) (Table 1). Finally, we found a trend of livers with visible Ito cells showing a larger frequency of fatty granulomas, compared with livers without visible Ito cells (*p* < 0.06).

In addition, we found one case of unifocal necrosis and a single case of fibrin exudation, described by Kelly (1993) and MacLachlan and Cullen (1995), but we did not investigate the significance further.

### Lipids.

**Figure 2:**
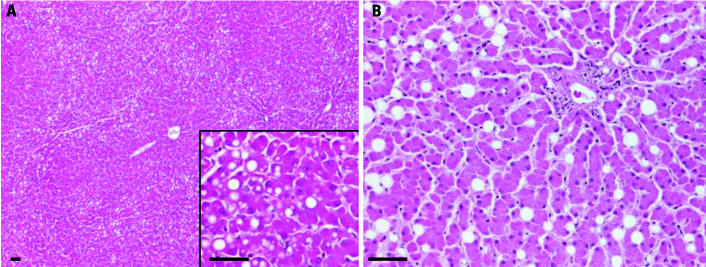
Lipid accumulation in liver tissue stained with H&E. (*A*) Zone 2–3 hepatocytic macrovesicular lipid (vacuoles; 2.5×) in a 4-year-old (subadult) female; inset, taken from (*A*; 10×). (*B*) Ito cell lipid accumulation in a 20-year-old female; 10×. Bars = 25 μm.

All animals showed hepatocytic microvesicular lipid accumulation (foamy cytoplasm), and 84% showed sharply demarcated macrovesicular lipid vacuoles in mainly periacinar (zones 2–3) hepatocytes (Table 1, Figure 2). In addition, we found non-parenchymal lipid vacuoles of diverging size and numbers in centroacinary Ito cells—located in the narrow space of Disse, between hepatocytes—mainly periacinary (zones 2–3) (Table 1, Figure 2) (Kelly 1993; Leighton et al. 1988; MacLachlan and Cullen 1995; Senoo et al. 1999, 2001). Intrahepatocytic lipid accumulation was not related to age (*p* > 0.05), whereas Ito cell lipid accumulation was highly related to age (*p* < 0.01) (Table 1). None of the lipid changes was related to sex or season (summer vs. winter) (Table 1).

### Bile duct proliferation and portal fibrosis.

**Figure 3:**
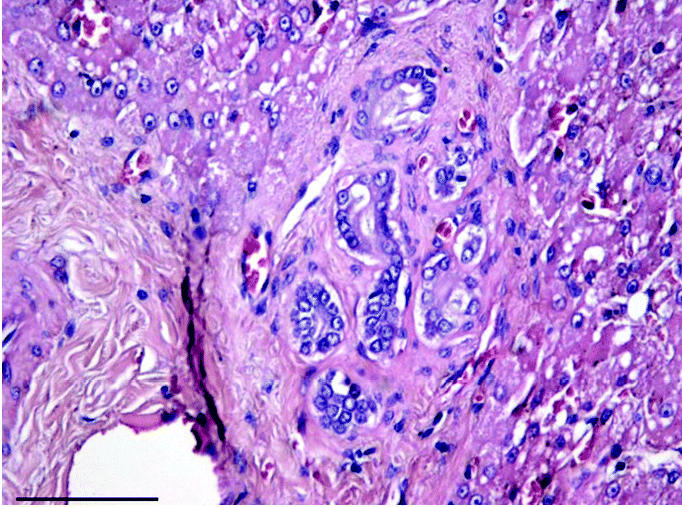
Mild bile duct proliferation accompanied by portal fibrosis (H&E; 20×). Bar = 50 μm.

Mild bile duct proliferation accompanied by portal fibrosis was found in 8% of the animals (Table 1, Figure 3). These changes were associated with age (both, *p* < 0.01); no relationships were found to sex or season (Table 1).

### OHCs and histologic changes.

Levels of ∑PCB, ∑CHL, ∑DDT, dieldrin, ∑HCH, HCB, and ∑PBDE in 65 of the examined polar bears are presented in Table 2. ∑CHL, ∑PCB, ∑DDT, dieldrin, ∑HCH, and ∑PBDE did not differ significantly among age/sex groups, but HCB was higher in subadults when compared with adult males (*p* ≤ 0.05) (Table 2). We found a significant negative relationship between age and HCHs, HCB, and dieldrin (all, *p* < 0.05) for adult females, and between age and ∑CHL and dieldrin in adult males (both, *p* < 0.01) (Table 2). Further information about age and sex variation of OHCs in the present East Greenland polar bears has been published by Dietz et al. (2004) and Sandala et al. (2004).

The statistical analyses were performed separately on subadults, adult females, and adult males in cases of ∑CHL, dieldrin, ∑HCH, and HCB because concentrations and/or age relationships differed between the three groups of individuals (Table 2). We tested whether the concentrations of each OHC group differed between the degree of histologic liver changes (absent vs. present); for adult females we found a significant relationship between ∑HCH and hepatocytic macrovesicular lipids (vacuoles), and for adult males we found a significant relationship between HCB and lipid granulomas (both, *p* < 0.05) (Table 3).

## Discussion

We found nuclear displacement toward the cell membrane in all individuals. In studies of polar bears from Svalbard, Sato et al. (2001) revealed the same findings. It has been proposed that this displacement is related to the high vitamin A accumulation (natural storage) in Ito cell cytoplasmic lipid droplets and hepatocytes, accumulated through the extensive feeding on blubber from ringed seal and bearded seal (Käkelä et al. 1997; Ramsay and Stirling 1988). In general, such a displacement is associated with hepatitis, carcinomas, hyperplasia (adenomatous), or regeneration (Sato et al. 2001). However, such changes were not found in the Svalbard study (Sato et al. 2001), and in only two cases were unifocal hepatitis and regeneration found in the present study. We could not evaluate whether there was a relation between nuclear displacement and OHCs or hepatocytic lipid accumulation because we found the displacement in nearly all individuals. Therefore, we hypothesize that displacement may be a natural phenomenon in free-ranging polar bears, probably related to vitamin A intake and/or a result of lipid/OHCs accumulation (cytoskeletal displacement).

### Mononuclear cell infiltrations and lipid granulomas.

Mononuclear cell infiltrates—accompanied by fibrosis—is a reaction to local depositioning of microorganisms and/or injury of local blood vessels from, for example, toxic compounds (Kelly 1993; MacLachlan and Cullen 1995). These cell infiltrates are therefore a nonspecific inflammatory reaction that can be linked to even minor tissue damage (Kelly 1993; MacLachlan and Cullen 1995). The fact that liver tissue, rich in visible Ito cells, had a higher number of lipid granulomas indicates that microorganisms (originating from the blood supply) play a role in the random multifocal necrosis (rupture of Ito cells) observed (Kelly 1993; MacLachlan and Cullen 1995). However, if the lipophilic toxic OHCs accumulate in the lipid rich Ito cells, we hypothesize that OHCs may play a role in the burst of Ito cells, as well.

### Lipids.

In the present study, we found macrovesicular lipid in periacinar hepatocytes. Because polar bears are hyperphagic from April to July, they build up their fat deposits during this period (Messier et al. 1992; Ramsay and Stirling 1988), and a seasonal pattern in Ito cell numbers may be expected as was the case for the fatty tissue lipid percentage (Dietz et al. 2004). Intrahepatocytic accumulated lipid vacuoles showed a zonary pattern similar to that found in individuals exposed to toxic substances, which produce a characteristic periacinar injury due to the low oxygen gradient (hypoxia and high concentrations of, for example, cytochrome P450). This could sensitize the liver parenchyma in this zone to metabolic disorders, resulting in lipid accumulation (Kelly 1993; MacLachlan and Cullen 1995; Parkinson 1996).

We also found lipid accumulation in periacinary Ito cells. In polar bears, the Ito cells are one of the major accumulation and storage sites for lipophilic vitamin A (Leighton et al. 1988; Senoo et al. 1999, 2001) and probably also lipophilic OHCs, as mentioned above. As for hepatocytic lipid accumulation, we did not find a seasonal pattern in the number of Ito cells, but we did find that the number of Ito cells is related to age. If the Ito cell number reflects the vitamin A exposure through marine prey species, mainly ringed seal and bearded seal (Ramsay and Stirling 1988), young bears would have lower numbers of Ito cells because they do not start eating prey rich in vitamin A until they are weaned at approximately 2 years of age (Derocher and Stirling 1994). This may then explain the age difference in the number of Ito cells in the liver.

### Bile duct proliferation and portal fibrosis.

Bile duct proliferation has been associated with toxic injury, parasitism, or periductular fibrosis in terrestrial animals (Kelly 1993; MacLachlan and Cullen 1995) and is therefore a non-specific reaction to chronic extrinsic and/or environmental factors. Specifically in arctic mammals, bile duct proliferations have been reported in arctic beluga whale (*Delphinapterus leucas*), but the pathogenesis of this could not be determined (Woshner et al. 2002).

Age-related portal fibrosis, due to chronic infections (cholangitis and biliary obstruction), is a common nonspecific histologic diagnosis in mammals (Kelly 1993; MacLachlan and Cullen 1995), and it has been reported in the Romanian brown bear (*Ursus arctos*) (Prunescu et al. 2003) and arctic beluga whale (Woshner et al. 2002). Prunescu et al. (2003) showed seasonal liver fibrosis (highest in spring) of the hepatic venous system, possibly due to pre-hibernation physiologic adaptations. Our findings were not in agreement with such a seasonal fibrosis pattern, however, because portal fibrosis was present with bile duct proliferations in all individuals.

### Liver changes and OHCs.

To our knowledge, liver histology in relation to environmental levels of OHCs has been studied only in birds, such as cormorants (Fabczak et al. 2000), and fish, such as common bream (Koponen et al. 2001), but never in marine or terrestrial mammals. Therefore, it is difficult to evaluate the relationship between liver histology and chronic exposure to environmental levels of OHCs in the East Greenland polar bear because basic knowledge in this field is extremely sparse.

Mononuclear cell infiltrates (lymphocytes and neutrophils) randomly distributed (lipid granulomas) or portally (around triads) have been associated with subacute PCB exposure in mink (*Mustela vison*) (Bergman et al. 1992). We found the same pattern in polar bears, which supports the hypothesis that OHCs could be a cofactor in the liver changes of the East Greenland polar bears in the present study. However, this could also be a result of microorganisms. Although the results from the laboratory studies are nonspecific reactions, parallels to our results are obvious.

Hepatotoxic substances (e.g., copper, pyrrolizidine alkaloids, carbon tetrachloride, and phytotoxins) usually produce a periacinar zone 2–3 injury due to the low oxygen gradient (hypoxia) and high concentrations of, for example, cytochrome P450 isozymes (activation of reactive metabolites) of this zone (Kelly 1993; MacLachlan and Cullen 1995; Parkinson 1996). We found such a zonary appearance in hepatocytic accumulation in the polar bears in the present study. Abnormal amounts of fat are known to be accumulated in the liver during high lipid ingestion, starvation, abnormal hepatocytic function, excessive dietary intake of carbohydrates, and decreased synthesis of -apoproteins (lipoproteins) (Kelly 1993; MacLachlan and Cullen 1995; Parkinson 1996). Hence, the large content of lipids in polar bear livers could be a function of hyperphagia and starvation due to seasonal changes in food resources, as discussed above, although we did not find a seasonal pattern. However, acute toxic investigations of PCBs, DDTs, and dieldrin in laboratory rats have shown to induce high lipid accumulation—probably due to decreased production of lipoproteins through impaired ATP synthesis and protein synthesis—in periacinary hepatocytes (accumulated as foamy cytoplasm or large vacuoles) (Bergman et al. 1992; Bruckner et al. 1974; Kelly 1993; Kimbrough et al. 1971, 1972; MacLachlan and Cullen 1995; Parkinson 1996). Therefore, OHCs may be a cofactor in the development of lipid accumulation in the present study, although significant differences in OHC concentrations were not found.

The signs of chronic inflammation, also in relation to Glisson’s triads (bile duct proliferation accompanied by portal fibrosis), as well as the hepatocytic lipid accumulation, could possibly indicate long-term exposure to liver toxic substances (OHCs) in the East Greenland polar bear, as well. However, other than the OHC considerations and age, liver histology in free-ranging Atlantic bottlenose dolphin (*Tursiops truncatus*) (Rawson et al. 1993) and arctic beluga whale (Woshner et al. 2002), in relation to mercury exposure, have shown changes similar to those in the present study. The East Greenland polar bears in the present study have also accumulated considerable amounts of mercury in the liver tissue (2.13–13.4 μg/g wet weight) (Dietz et al. 1990, 2000), which are in the range of adverse toxic effect levels for terrestrial mammals (Thompson 1996).

## Conclusions

In the present study, we found the following histologic changes in liver tissue from 79 East Greenland polar bears: nuclear displacement, mononuclear cell infiltrations, mild bile duct proliferation accompanied by portal fibrosis, and fat accumulation. Two of the changes (Ito cells and bile duct hyperplasia accompanied by portal fibrosis) were related to age, whereas none were related to sex or season. The signs and type of chronic inflammation, and the zonary lipid accumulation in hepatocytes, may indicate chronic exposure to environmental levels of OHCs. In addition, we found significant relationships for ∑HCH and hepatocytic lipid accumulation in adult females and between HCB and lipid granulomas in adult males. We therefore suggest that the histologic changes were a result of aging and long-term exposure to OHCs, but other environmental factors, such as microorganisms and mercury, cannot be excluded.

## Correction

The range of mercury in liver tissue of East Greenland polar bears was incorrect in the original manuscript published online but has been corrected here. The authors also found additional information that was not included in their original manuscript: Hori et al. [Hori S, Obana H, Kashimoto T, Otake T, Nishimura H, Ikegami N, et al. 1982. Effect of polychlorinated biphenyls and polychlorinated quaterphenyls in cynomolgus monkey (*Macaca fascicularis*). Toxicology 24(2):123–139] found an association between bile duct proliferation and PCB exposure, and also reported that mononuclear cell infiltrates were associated with subacute PCB exposure in cynomolgus monkeys (*Macaca fascicularis*). Also, the authors would like to state that it is impossible to evaluate whether liver changes and possible demineralization of the skeletal system (Sonne et al. 2004) and renal lesions (Sonne et al., in press) have an impact on the health status of each individual polar bear.

## Figures and Tables

**Table 1:** Prevalence of histologic liver changes in relation to age, sex, and season in 79 East Greenland polar bears sampled during 1999–2002.

	Degree of change [% (*n*)]						
Histologic liver change	Absent	Mild	Moderate	Severe	Age [(*F* )*p*]	Sex [ (*F* )*p*]	Season [(*F* )*p*]
Portal mononuclear cell infiltrations	82 (65)	8 (6)	8 (6)	2 (2)	NS	NS	NS
Random mononuclear cell infiltrations	87 (69)	11 (9)	1 (1)	0 (0)	NS	NS	NS
Hepatocytic intracellular fat	0 (0)	16 (13)	24 (19)	60 (47)	NS	NS	NS
Lipid granulomas	24 (19)	35 (28)	32 (25)	9 (7)	NS	NS	NS
Lipid accumulation in Ito cells	25 (20)	18 (14)	24 (19)	33 (26)	(8)*	NS	NS
Mild bile duct hyperplasia with fibrosis	92 (73)	8 (6)	0 (0)	0 (0)	(11)*	NS	NS

NS, not significant. Hepatic changes are divided into degrees of change (absent, mild, moderate, and severe); see “Materials and Methods” for criteria.

* Individuals with histologic liver changes were significantly older (mean age) than individuals without histologic liver changes (*p* < 0.01).

**Table 2:** OHC concentrations (mean ± SD, ng/g lipid weight) in subcutaneous adipose tissue of 65 East Greenland polar bears investigated for histologic liver changes during 1999–2001.

OHCs	Subadults (*n* = 27)	Adult females (*n* = 21)	Adult males (*n* = 17)
∑PCB	6,130 ± 3,290	5,303 ± 2,157	7,081 ± 3,197
∑DDT	468 ± 240	380 ± 206	476 ± 259
∑CHL	1,518 ± 1,009	1,349 ± 559	1,016 ± 576*
Dieldrin	215 ± 114	179 ± 59**	172 ± 93#
∑HCH	184 ± 73	182 ± 155##	217 ± 144
HCB	114 ± 103	75 ± 68†	51 ± 32††
∑PBDE	57 ± 32	59 ± 36	51 ± 32

* Significant negative relationship with age (*p* < 0.01; *R*^2^ = 0.51).

** Significantly negative relationship with age (*p* ≤ 0.05; *R*^2^ = 0.26).

# Significant negative relationship with age (*p* < 0.01; *R*^2^ = 0.45).

## Significantly negative relationship with age (*p* ≤ 0.05; *R*^2^ = 0.25).

† Significantly negative relationship with age (*p* ≤ 0.05; *R*^2^ = 0.2).

†† Significantly lower compared with subadults (*p* ≤ 0.05).

**Table 3:** Significant results from analyses of relationships between histologic liver changes and OHCs in adult female and male East Greenland polar bears, 1999–2001.

Age/sex group	Histologic liver change	OHCs	(*n*, *F*, *R*^2^)*p*
Adult females	Hepatocytic intracellular fat	∑HCH	(17, 8.5, 0.42)*
Adult males	Lipid granulomas	HCB	(21, 9.8, 0.52)**

* Significantly higher OHC level (least square mean) in individuals with mild/moderate changes than in individuals without changes (*p* ≤ 0.05).

** Significantly higher OHC level (least-square mean) in individuals with mild/moderate changes than in individuals without changes (*p* < 0.01).
